# The Proximal Humerus Outcome Score at One Year (POSY) Predicts Which Patients Have Poor Functional Outcomes Following Operative Fixation of Proximal Humerus Fractures

**DOI:** 10.7759/cureus.26631

**Published:** 2022-07-07

**Authors:** Nina D Fisher, Adam Driesman, Hesham Saleh, Kenneth A Egol, Sanjit R Konda

**Affiliations:** 1 Orthopedic Surgery, New York University (NYU) Langone Health, New York City, USA; 2 Orthopedic Surgery, Jamaica Hospital Medical Center, New York City, USA

**Keywords:** geriatric fracture, predictive scoring, operative repair, functional outcomes, proximal humerus fracture

## Abstract

Background: The ability to predict long-term outcomes following surgical fixation of proximal humerus fractures would help identify patients at risk of poor functional outcomes. The purpose of this study was to develop a simple score based on preoperative data that can accurately predict functional outcomes for patients following operative management of proximal humerus fractures.

Methods: Over a 12-year period, all proximal humerus fractures surgically treated with a locked proximal humerus plate at a single institution were enrolled in a prospective database. Inclusion criteria in this analysis were any patient with a minimum of a one-year functional outcome score. Patients were assigned to the poor outcome cohort if their Disabilities of the Arm, Shoulder, and Hand (DASH) score at that time point was greater than 10 points above the mean DASH score. Logistic regression was used to build a predictive formula for cohort membership using p < 0.15 and an area under the receiver operator characteristic curve (AUROC) value was calculated to define the overall predictive capacity.

Results: A total of 165 patients with an average age of 60.91±13.5 years met the inclusion criteria, with 47 (28.5%) patients assigned to the poor outcome group and 118 (71.5%) patients assigned to the good outcome group. Older age (p = 0.088), BMI (p = 0.019), age-adjusted CCI (p = 0.001), non-Caucasian race (p = 0.017), no college degree (p < 0.0005), unemployed (p < 0.0005), and worker’s compensation case (p = 0.002) were found to be significant predictors of poorer outcome and were used to create a final formula through logistic regression which predicted the probability of a poor outcome (Nagelkerke R Square = 0.403; Hosmer and Lemeshow = 0.902; AUROC = 0.839 [CI: 0.762-0.917]). Once each patient was assigned a score, cutoff values were defined that divided the cohort into three groups. High-risk patients had a score above 50%, and 19 (73.1%) of these patients had a poor outcome.

Conclusions: The POSY score is a tool that can predict the functional outcome at one year or greater following surgical intervention for a proximal humerus fracture. Patients who score above 50% are considered at high risk for a poor functional outcome. In the era of value-based care, the POSY score may be used to direct resource utilization while improving outcomes.

## Introduction

Fractures of the proximal humerus are bi-modal with two primary mechanisms seen: low-energy falls in older patients and high-energy accidents in younger patients [1,2]. Proximal humerus fractures are considered a fragility fracture when seen in the elderly and have an increasing incidence rate in older individuals (in particular in older women) secondary to osteoporosis [1-3]. As the population continues to increase in size and age, proximal humerus fractures will become a greater burden on the healthcare system [3]. Management of proximal humerus fractures has been highly debated in the literature in recent years. There is debate in the literature as to how these fractures should be managed, both in terms of operative versus non-operative treatment and type of surgical intervention if operative treatment is indicated. Surgical options include tension bands, percutaneous pinning, intramedullary nailing, locked plates, hemiarthroplasty, and reverse total shoulder arthroplasty [1,4,5]. The use of a locking plate offers several advantages in that it provides good functional outcomes with a relatively low complication rate, and can overcome the challenges of treating fractures in patients with poor bone quality [6-8]. Although some studies report higher complication rates following treatment with locked plates, this is usually attributed to an inadequate reduction, more complex fracture pattern, or possible inexperience of the surgeon [9,10].

Patient-reported outcomes are used to evaluate outcomes of treatment as well as the success of surgical procedures [11-14]. There are many studies that examine short-term outcomes such as postoperative complications, length of hospital stay, disposition, and mortality, yet few studies examine preoperative characteristics about long-term outcomes [15,16]. However, if orthopedic surgeons could identify patients preoperatively at risk of having a poor functional outcome, they could perhaps indicate these patients for different surgical treatments (e.g., hemiarthroplasty versus reversed total shoulder arthroplasty) or an early intervention postoperatively regardless of surgical fixation. The purpose of this study was to develop a simple score using pre-operative data that could be used to accurately predict a patient’s functional outcomes at 1 year following surgical fixation for a proximal humerus fracture ORIF.

## Materials and methods

All proximal humerus fractures that were surgically treated with a locking plate at two institutions within one academic medical center were enrolled in an IRB-approved database and prospectively followed over a 12-year period. Inclusion criteria were any patient over the age of 18 years who sustained a proximal humerus fracture, underwent surgical treatment with a locked plate, and had a 12-month follow-up available. Patients with less than 12-month follow-up were excluded from the analysis. All patients were surgically treated in a similar manner previously described by the senior author [17]. Demographic information including age, gender, race, education level, employment status, and other co-morbidities was collected pre-operatively, and initial injury radiographs were classified by the AO/OTA and Neer classification systems [18,19]. Baseline functional assessment was evaluated using the Disabilities of the Arm, Shoulder, and Hand (DASH) patient-reported outcome measure [20]. The DASH is intended to measure shoulder, elbow, wrist, and hand function in one metric on a scale of 0 to 100, with 0 being the best possible score (complete functional ability) and 100 being the worst possible score (no functional ability), and has been rigorously correlated with shoulder-specific measures [12]. The DASH is the most validated measure of upper extremity functional status and has been shown to strongly correlate with pain levels [13]. Patients were all sent for standard physical therapy post-operatively. Patients were examined using radiographic evaluations and the DASH at 3, 6, 12, and greater than 12 months. All postoperative complications were recorded as they occurred.

Patients were assigned to the poor outcome cohort if their DASH score at 12+ month follow-up was 10 points above the mean DASH score of the entire cohort [21]. The poor outcome and good outcome groups were statistically compared over a number of variables to determine which factors were significantly different between groups. All statistical analysis was conducted using SPSS Version 20 (SPSS, Inc., Chicago, IL, USA). The Mann Whitney U test was used for continuous variables and Pearson’s Chi-Square was used for categorical variables. A p value of less than 0.15 was considered significant for inclusion in regression analysis. Variables that were significantly different between groups were included in a logistic regression, which was used to build a predictive formula for group membership. An area under the receiver operator characteristic curve (AUROC) value was calculated to define the overall predictive capacity.

## Results

One hundred and sixty-five patients with complete data at 12 months or greater postoperatively were included in this analysis, with a mean DASH score for all patients of 22.23±21.8. Forty-seven (28.5%) patients were assigned to the poor outcome group and 118 (71.5%) patients were assigned to the good outcome group. The poor and good outcome cohorts were significantly different in terms of age, body mass index (BMI), age-adjusted Charlson Co-morbidity Index (CCI), Caucasian race, and education level (defined as a binomial variable in terms of whether or not the patient possessed a college degree), employment status, and worker’s compensation case (Table 1).

**Table 1:** Patient demographics DASH - Disabilities of the Arm, Shoulder, and Hand; CCI - Charlson Co-morbidity Index

	Total (N = 165)	Good Outcome (N = 118)	Poor Outcome (N = 47)	Sig.
1 Year DASH	22.32±21.8	10.77±9.1	52.87±16.3	---
Follow-up Interval (Months)	19.29±13.6	21.06±15.2	16.05±7.6	---
Age at Injury	60.91±13.5	59.54±14.5	65.38±11.4	0.088
BMI	28.24±7.1	27.04±6.7	29.70±7.2	0.019
Age Adjusted CCI	3.12±1.7	2.84±1.7	3.87±1.0	0.001
Caucasian Race	76.4% (126)	81.4% (96)	63.8% (30)	0.017
Female Gender	67.9% (112)	65.3% (77)	74.5% (35)	0.253
College Degree (N = 142)	56.3% (80)	69.0% (69)	26.2% (11)	< 0.0005>
Married (N = 159)	53.5% (85)	56.3% (63)	46.8% (22)	0.276
Employed (N = 147)	45.6% (67)	55.2% (58)	21.4% (9)	< 0.0005>
Worker’s Compensation Case (N = 153)	4.9% (6)	0.9% (1)	11.6% (5)	0.002
Current Smoker (N = 160)	18.1% (29)	19.1% (22)	15.6% (7)	0.598
AO/OTA Classification				0.788
	11-A: 25.5% (42)	11-A: 26.3% (31)	11-A: 23.4% (11)	
	11-B: 38.2% (63)	11-B: 39.0% (46)	11-B: 36.2% (17)	
	11-C: 36.4% (60)	11-C: 34.7% (41)	11-C: 40.4% (19)	
Neer Classification				0.259
	2-part: 24.2% (40)	2-part: 27.1% (32)	2-part: 17.0% (8)	
	3-part: 56.4% (93)	3-part: 55.9% (66)	3-part: 57.4% (27)	
	4-part: 19.4% (32)	4-part: 16.9% (20)	4-part: 25.5% (12)	

Variables related to the initial injury pattern, such as OTA and Neer fracture classification, were not significantly different between the two groups. The poor outcome group was on average older in age, and had higher average BMI and CCI. This cohort also had a lower proportion of patients who were of Caucasian race, had obtained a college degree, and were currently employed, and a higher proportion of worker’s compensation cases.

The seven predictor variables (age, BMI, age-adjusted CCI, Caucasian race, college degree, employment, and worker’s compensation status) that were statistically significantly different between the poor and good outcome cohorts were included in a binomial logistic regression (Table 2).

**Table 2:** Logistic regression predicting poor outcome group membership

	Sig.	Odds Radio	95% CI for Odds Radio	
Age	0.576	0.984	0.928	1.042
BMI	0.480	1.027	0.954	1.106
Age Adjusted CCI	0.128	1.430	0.903	2.267
Caucasian Race	0.125	2.419	0.783	7.473
College Degree	0.017	3.827	1.271	11.519
Employed	0.087	3.023	0.851	10.737
Worker’s Compensation Case	0.021	18.314	1.557	215.431
Constant	0.042	0.027	---	---

Only college degree and worker’s compensation were statistically significant within the equation. However, employment status was trending towards significance. Odds ratios were also calculated within the logistic regression (Table 2). Membership in the poor outcome group (based on DASH scores) was 2.5 times more likely for patients not of Caucasian race, 3 times more likely for patients who were unemployed, and 4 times more likely for patients without a college degree. Worker’s compensation cases were 18 times as likely to have a poor outcome, yet this prediction may be skewed by the relatively low number of worker’s compensation present in our analysis.

Overall the probability of a bad outcome using the POSY score was statistically significant (Hosmer and Lemeshow = 0.902, Nagelkerke R2 = 0.403) and correctly predicted 79.5% of cases.

The POSY score sensitivity was 51.4% and specificity was 91.1%. Of all cases predicted to be in the poor outcome group, 70.4% were correctly predicted (positive predictive value). Of all cases predicted to be in the good outcome group, 82.0% were correctly predicted (negative predictive value). There were three outliers identified that were not eliminated from the analysis. The area under the receiver operator curve (AUROC) was 0.839 with 95% confidence interval of 0.762 to 0.917.

**Figure 1:**
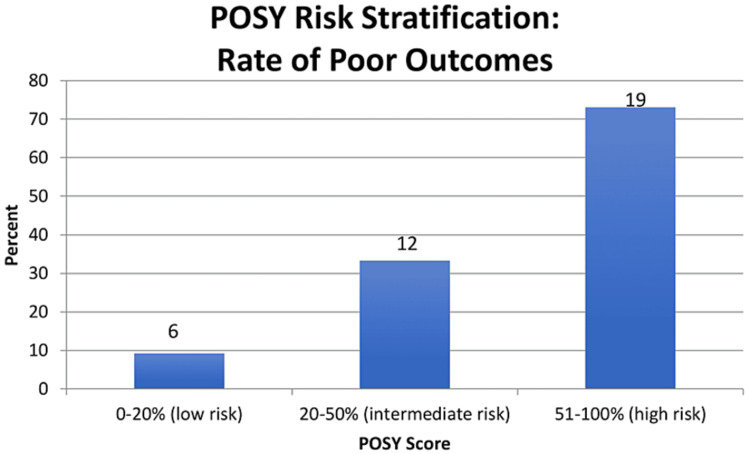
POSY risk stratification

Two cut-off values (20% and 50%) were identified that divided the patients into three groups based on their POSY score (Figure 1). Below 20% (low risk), 9.2% of patients had a poor outcome. Greater than 20% and less than 50% (intermediate risk), 33.3% of patients had a poor outcome. 50% and above (high risk), 73.1% of patients had a poor outcome.

While the POSY score was calculated using pre-operative factors, there was a higher rate of complications in the poor outcome cohort than the good outcome cohort. Seventeen (36.2%) patients in the poor outcome cohort experienced a total of 21 complications, with four (23.5%) patients experiencing multiple complication. Comparatively, 21 (17.8%) patients in the good outcome group experienced a total of 22 complications, with one patient experiencing multiple complications. In the poor outcome cohort, there was a 23.8% (5/21) rate of osteonecrosis, and a 19.0% (4/21) screw penetration rate. There was a lower rate (13.6%, 3/22) of osteonecrosis in the good outcome cohort but a higher rate (54.5%, 12/22) of screw penetration.

## Discussion

Although indications for operative fixation are debated within the literature, operative fixation of proximal humerus fractures with a locking plate has demonstrated good functional outcomes [6-9]. However, there remains a range of functional recovery in patients who sustain a proximal humerus fracture, and it is valuable to be able to identify patients at risk for a poor outcome. The purpose of this study was to determine patient characteristics that are associated with a poor functional outcome following open reduction internal fixation with a locking proximal humerus plate. The risk factors identified through this analysis were older age, higher BMI or CCI, non-Caucasian race, a lower education level (i.e., less than a college degree), unemployment status, and worker’s compensation case. All are factors that can be identified by the treating physician and thus affect treatment management. Additionally, the identified risk factors are all intrinsic patient characteristics, suggesting that the initial fracture pattern does not affect functional outcomes following proximal humerus fractures [17].

The risk factors identified through this analysis are all factors that have been previously reported as contributing to negative outcomes following orthopedic surgery. Modifiable risk factors include BMI, age-adjusted CCI, education level, and employment status. Increased BMI and/or obesity have been shown to increase postoperative complications in proximal humerus fracture patients, yet few studies report on the long-term effects [22]. Yian et al. have reported that CCI correlates with 1-year mortality following proximal humerus fracture, suggesting that certain co-morbidities can affect outcomes with this fracture pattern [15]. Patients’ socioeconomic status, which includes education level and employment status, has been shown to significantly influence outcomes following orthopedic surgery [23,24]. Non-modifiable risk factors included age, race, and worker’s compensation status. Patients who are 65 years and older have been previously reported to have decreased functional abilities based on DASH scores reported one-year postoperatively from surgical treatment for proximal humerus fractures [7]. Racial disparities following orthopedic surgery are well-documented in the literature, yet studies usually focus on arthroplasty patients [25]. The most significant predictor of a poor outcome was the worker’s compensation case. Previous studies have reported that patients receiving worker’s compensation have a two-fold increase in negative outcomes following orthopedic surgery [26]. Our study reports a high risk of a poor outcome associated with worker’s compensation and of the six patients who were worker’s compensation cases, five were members of the poor outcome cohort with an average DASH score of 56.2±17.8 at one year.

Functional outcome scores are typically better in patients without complications, so it is expected that the poor outcome cohort would have a greater incidence of complications [8]. The need for revision surgery following surgical fixation for proximal humerus fractures has been shown to be a predictive factor for unsatisfactory results [27]. Therefore, it is possible that some of the patients in the poor outcome cohort demonstrated worse functional outcomes as a result of re-operation secondary to complications such as screw penetration, which often requires removal of hardware or conversion to total shoulder arthroplasty, and osteonecrosis of the humeral head, which is often converted to arthroplasty [28,29]. Osteonecrosis of the humeral head is a painful and debilitating condition that leads to decreased function of the shoulder joint [29,30]. The higher rate of osteonecrosis within the poor outcome cohort may have also contributed to their worse functional outcome scores. 

This analysis is limited by the definition of a poor outcome. Although 10 points above the mean DASH score is a reasonable estimation of a poor outcome, a larger threshold, i.e., 15 or 20 points above the mean DASH score, might increase the predictive nature of this equation. However, given the relatively small sample size, a 10-point limit was used to keep the groups relatively balanced. We also included patients of all ages and mechanisms of injury, and although our cohort was primarily of the older age demographic with low energy mechanisms, this could have also affected the analysis. Additionally, although all patients were prescribed standardized physical therapy, not all patients may have attended, yet this was not recorded in a systematic way and thus could not be included in the analysis. Furthermore, we only analyzed patients who had follow-up available at 1 year or greater. However, functional outcomes do not significantly change between 12 months and 24 months following major trauma, so we felt it was reasonable to compare all patients who had DASH scores available at one year or greater [31,32]. This study is also limited by the fact that this analysis was only conducted on patients who were operatively treated with locking plates. While it provides insight into the outcomes of proximal humerus fractures treated with locking plates, it does not reflect the outcomes of patients treated with other surgical options (i.e., arthroplasty) or non-operatively. In order to increase the applicability of this predictive analytic technique for proximal humerus fractures, this analysis should be replicated with proximal humerus fracture patients treated with arthroplasty or non-operatively. Furthermore, by predicting the functional outcomes of proximal humerus fracture patients, this score has the potential to compare treatment options for proximal humerus fractures and thus contribute to the current debate within the literature as to the best management of this injury.

## Conclusions

The POSY score tool is a predictor of functional outcome at one year or greater following surgical intervention for a proximal humerus fracture. Patients who score above 50% are considered at high risk for a poor functional outcome. Patients who have poor functional outcomes at one year postoperatively will usually demonstrate poor functional outcomes at longer follow-up intervals, so patients at risk for a poor functional outcome need to be identified as early as possible. These patients should either be considered pre-operatively for alternative treatment options or considered for non-operative care. These patients may also be indicated for more aggressive rehabilitation following surgical intervention. In the era of value-based care, the POSY score can be used to direct resource utilization while improving outcomes.
